# Prediction Models for Sepsis-Associated Thrombocytopenia Risk in Intensive Care Units Based on a Machine Learning Algorithm

**DOI:** 10.3389/fmed.2022.837382

**Published:** 2022-01-27

**Authors:** Xuandong Jiang, Yun Wang, Yuting Pan, Weimin Zhang

**Affiliations:** Intensive Care Unit, Dongyang Hospital of Wenzhou Medical University, Jinhua, China

**Keywords:** sepsis-associated thrombocytopenia, intensive care unit, machine learning, artificial intelligence, prediction

## Abstract

Sepsis-associated thrombocytopenia (SAT) is a common complication in the intensive care unit (ICU), which significantly increases the mortality rate and leads to poor prognosis of diseases. Machine learning (ML) is widely used in disease prediction in critically ill patients. Here, we aimed to establish prediction models for platelet decrease and severe platelet decrease in ICU patients with sepsis based on four common ML algorithms and identify the best prediction model. The research subjects were 1,455 ICU sepsis patients admitted to Dongyang People's Hospital affiliated with Wenzhou Medical University from January 1, 2015, to October 31, 2019. Basic clinical demographic information, biochemical indicators, and clinical outcomes were recorded. The prediction models were based on four ML algorithms: random forest, neural network, gradient boosting machine, and Bayesian algorithms. Thrombocytopenia was found to occur in 732 patients (49.7%). The mechanical ventilation time and length of ICU stay were longer, and the mortality rate was higher for the thrombocytopenia group than for the non-thrombocytopenia group. The models were validated on an online international database (Medical Information Mart for Intensive Care III). The areas under the receiver operating characteristic curves (AUCs) of the four models for the prediction of thrombocytopenia were between 0.54 and 0.72. The AUCs of the models for the prediction of severe thrombocytopenia were between 0.70 and 0.77. The neural network and gradient boosting machine models effectively predicted the occurrence of SAT, and the Bayesian models had the best performance in predicting severe thrombocytopenia. Therefore, these models can be used to identify such high-risk patients at an early stage and guide individualized clinical treatment, to improve the prognosis of diseases.

## Introduction

Artificial intelligence (AI) has enabled many cutting-edge scientific research achievements in the field of medical care, especially for acute and severe diseases. In fields such as disease risk assessment, early warning of disease deterioration, and early warning of death, AI can alert officials regarding potential risks earlier and more accurately. Machine learning (ML) is a branch of AI, and it has been used for predicting disease outcomes. Using the Medical Information Mart for Intensive Care (MIMIC) database, Garcia Gallo et al. (1) established a model to predict the mortality of patients with severe sepsis based on the ML algorithm, which achieved better evaluation results than traditional scoring systems such as Sequential (sepsis-related) Organ Failure Assessment (SOFA) Score and Simplified Acute Physiology Score II. Thorsen-Meyer et al. (2) applied the ML algorithm and further employed intensive care unit (ICU) time series data analysis to predict the 90-day mortality in real-time, thus improving the prognosis of diseases in ICU patients.

Sepsis-related thrombocytopenia (SAT) is a common complication in the ICU; in particular, the incidence of thrombocytopenia in patients with septic shock can be as high as 55% (3). SAT involves many mechanisms (4), which might include inflammation-mediated platelet production changes, endothelial dysfunction, abnormal blood coagulation function, and hemodilution. Thrombocytopenia can significantly increase the incidence of complications and mortality in patients with sepsis (5). A study by Azkárate et al. (6) showed that thrombocytopenia was associated with a 1.7-fold increased risk of mortality in severe sepsis patients. Thrombocytopenia may cause severe hemorrhage; a multicenter observational study (7) in UK ICU found that a total of 169 patients (9% of the study population) received platelet transfusion, and the prevalence of severe thrombocytopenia (<50 × 10(9) /L) was 12.4, and 35.4% of the patients finally died in the ICU. In actual clinical work, when a decrease in platelet count is observed for a patient, especially a severe decrease, platelets should be infused in time to reduce the risk of bleeding because platelets cannot be stored for a long time. However, patients may have to wait for 2–3 days from the beginning of platelet reservation to the actual infusion of platelets. In this process, the patients are at a high risk of bleeding and may even experience hemorrhagic shock, which is life-threatening. Early detection of platelet decrease is crucial for critically ill patients.

Presently, there are many related models for predicting sepsis using artificial intelligence (8, 9), which can enhance doctors' medical decision-making ability for patients with sepsis. However, research on predicting SAT and severe thrombocytopenia in the ICU is lacking, and effective models for predicting SAT using ML algorithms have not yet been established. Therefore, we used a large amount of real-time data from the ICU to establish prediction models for thrombocytopenia in ICU sepsis patients for the early identification of patients with a high risk of thrombocytopenia, which would help reduce the occurrence of bleeding events and improve the prognosis of diseases in patients.

## Materials and Methods

### Study Design and Research Subjects

Our study was reported according to the guidelines of the TRIPOD (10) statement (Checklist in Additional File 1). A retrospective study was conducted with 1,455 sepsis patients who were admitted to the ICU of Dongyang People's Hospital between January 1, 2015, and October 31, 2019. External validation was performed using the MIMIC III dataset (11), a freely accessible online critical care database. The inclusion criteria were age ≥18 years and admission to the ICU with sepsis. The exclusion criteria were patients who had hematological malignancy, cirrhosis patients who had underlying thrombocytopenia before ICU admission, and patients who had undergone splenectomy.

This study was approved by the Ethics Committee of Dongyang People's Hospital (DRY-2021-YX-178). The need for informed consent was waived because of the retrospective, observational study design. The data were anonymously analyzed after the removal of personal information from the data. One author (XJ) obtained permission for accessing the MIMIC database after the completion of “Protecting Human Research Participants,” an online training course launched by the National Institutes of Health (certification number: 7632299).

### Data Collection and Grouping

#### Data Collection

Data were collected using the medical record information mining software provided by Shanghai Le9 Healthcare Technology Co., Ltd. The collected information included the following: (1) basic clinicodemographic information [age, sex, disease severity (Acute Physiology and Chronic Health Evaluation, APACHE II score, SOFA score), smoking history, alcohol abuse history, and complications]; (2) blood gas, blood routine, biochemistry, and liver function indicators on the first day of ICU admission; and (3) clinical outcomes (mortality, time on ventilator, length of ICU stay, length of hospital stay, and hospitalization cost).

#### Diagnostic Criteria

Definition of SAT: Sepsis patients with thrombocytopenia.

Thrombocytopenia (12, 13):Platelet count <100 × 109/L or a 30% relative decrease of the baseline platelet count during ICU stay; the baseline platelet count was defined as the highest value over the past seven days before ICU admission. We used the initial platelet count in ICU admission as baseline platelet count for patients without platelet count measurement before ICU admission.

Severe thrombocytopenia (14, 15): Platelet count <50 × 109/L during ICU stay.

Sepsis 3.0 (16): Organ dysfunction triggered by an infection that endangers the patient's life and rapid increase in the SOFA score, with a total score of two points.

Sepsis shock (16): The patient with sepsis requiring vasopressors to maintain mean blood pressure at 65 mmHg or higher and having a serum lactate level higher than 2 mmol/L (18 mg/dL) after fluid resuscitation.

### Data Processing

#### Selection of Independent Variables

Sixty-five potentially related variables were preliminarily screened. After excluding three variables with more than 15% of missing values, the remaining 62 variables were subjected to data preprocessing using CARET in R language. Thirteen variables showing a strong correlation (correlation coefficient >0.9) with other independent variables were eliminated. The remaining 57 variables were then subjected to feature selection using the backward selection method, random forest (RF) sampling, and 10% cross-checking. Then, the efficiency (precision, recall, accuracy, and specificity, the cutoff point was 0.5) was calculated, and the variables were ranked according to their importance. The 10 most important variables were retained.

#### Handling of Missing Values

Variables with >15% missing values were deleted. If the incidence of missing values was <2%, the mean value of the variable was used to replace the missing values. The missing values of variables with loss rates of >2 and <15% were replaced using multiple imputations.

#### Handling of Outliers

Outliers were detected using the interquartile range (IQR), i.e., the difference between the upper and lower quartiles of the boxplot. We used 1.5 times of IQR as the standard, and points exceeding this criterion (the upper quartile + 1.5 times of IQR, or the lower quartile – 1.5 times of IQR) were defined as outliers. The excluded outliers were handled as missing values.

#### Model Establishment

The following R packages for the ML method were used: caret, ipred, ranger, arm, nnet, and gbm. Samples were randomly divided into training set and test set in a 7:3 ratio. All ML models were evaluated using 10× cross-validation.

The hyperparameters were adjusted by grid search as follows. For the RF model, the number of trees and mtry parameters were adjusted. For the neural network (NNET) model, size and decay parameters were adjusted. For the gradient boosting machine (GBM) model, n.trees, interaction.depth, and shrinkage were adjusted. Finally, the importance of variables was sorted using the function “varImpPlot” within the “caret” package in *R*.

##### Model Validation and Evaluation

The area under the receiver operating characteristic curve (AUC), sensitivity, specificity, and 95% CI of each model were calculated. The confusion matrix was evaluated using accuracy, precision, and recall as parameters presented in Table 1. Local Interpretable Model-Agnostic Explanations (LIME) provides another method for model interpretation (17).

**Table 1:** Comparison of the additional evaluation metrics of four machine learning models in external validation.

**Models for predicting thrombocytopenia**				
	**RF**	**Bayesian**	**NNET**	**GBM**
Accuracy	0.61	0.55	0.68	0.67
Precision	0.61	0.59	0.71	0.67
Recall	0.72	0.40	0.65	0.74
Specificity	0.50	0.70	0.71	0.61
**Models for predicting severe thrombocytopenia**				
	**RF**	**Bayesian**	**NNET**	**GBM**
Accuracy	0.71	0.68	0.72	0.72
Precision	0.47	0.45	0.48	0.48
Recall	0.55	0.84	0.59	0.49
Specificity	0.77	0.62	0.77	0.81

*RF, random forest; NNET, neural network; GBM, gradient boosting machine*.

#### Statistical Analysis

Descriptive statistics were analyzed conventionally using the CBCgrps package in R (18). Normally distributed measurement data were expressed as x ± s and compared between groups using the two-independent-samples *t*-test. Meanwhile, non-normally distributed data were expressed as M (P25, P75) and compared using the Mann–Whitney *U* test. Enumeration data were expressed in terms of the rate and percentage and compared between the groups using the χ^2^ test. All statistical analyses were performed using R (software version 3.6.3). A *P*-value of 0.05 was considered significant.

## Results

### Comparison of Basic Information and Clinical Outcomes

**Figure 1:**
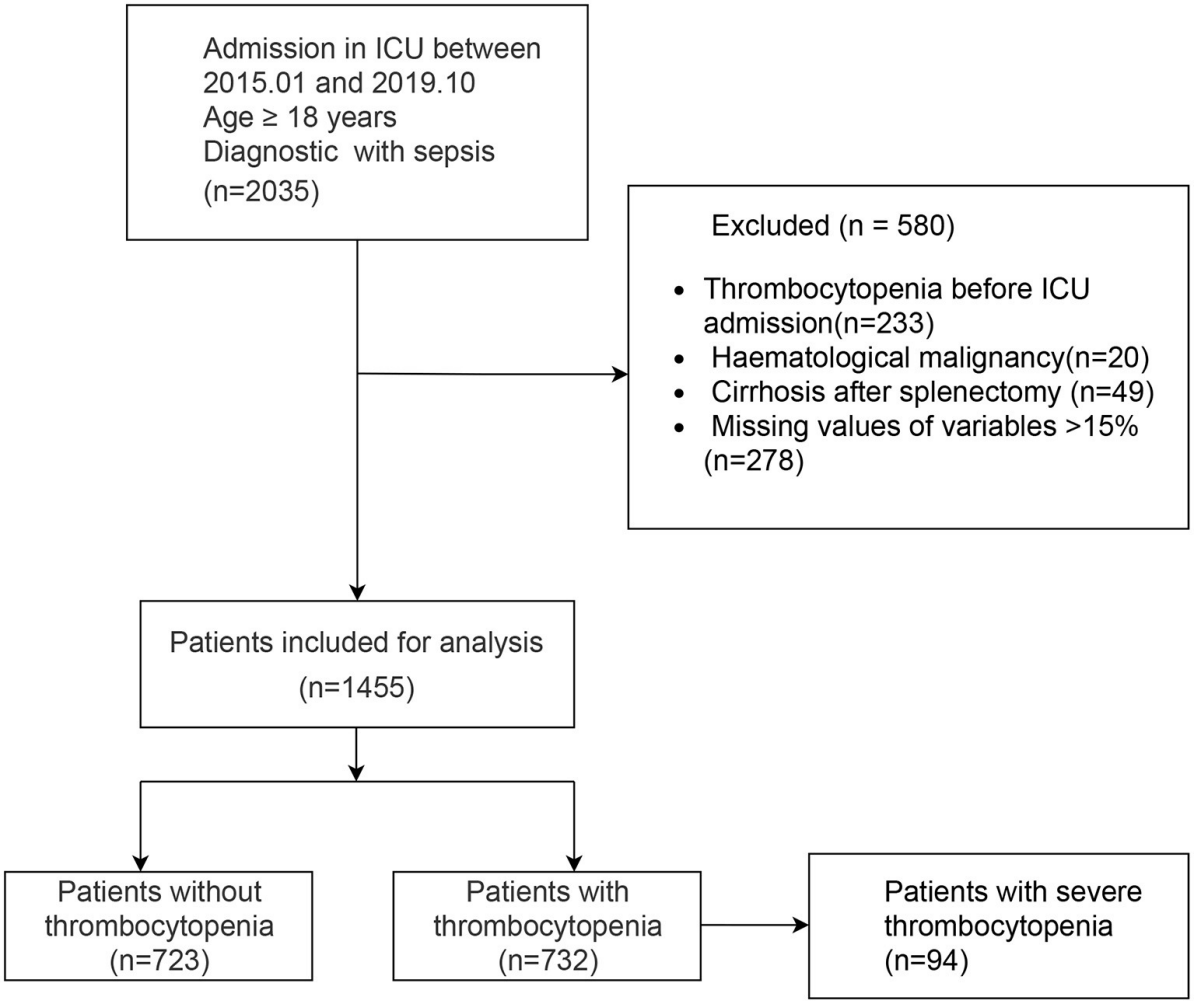
Flow chart of the study. ICU, Intensive Care Unit.

A total of 1,455 patients with sepsis were included in this study. The flow chart of the study is shown in Figure 1, including 732 SAT patients (49.7%). Regarding the sources of infection, pulmonary infection accounted for the highest proportion, with 1,019 cases (70%), followed by blood-borne infection, with 217 cases (14.9%), and urinary tract infection, with 132 cases (9.1%). There were 189 patients with septic shock, and 76.7% of them had SAT.

Table 2 shows a comparison of general clinical information and clinical outcomes between the thrombocytopenia and non-thrombocytopenia groups. There was no significant difference in age and gender between the two groups, with an average age of 65.6 ± 16.6 years and 63.6% of subjects being male. The disease conditions in the thrombocytopenia group were more serious, and the APACHE and SOFA scores were significantly higher than those in the non-thrombocytopenia group, with statistically significant differences (*P* < 0.001). There were significant differences in terms of mechanical ventilation time, length of ICU stays, length of hospital stays, and mortality between the two groups (*P* < 0.001), and the clinical outcome of the thrombocytopenia group was worse. Table 3 shows a comparison of infection site and clinical outcomes between the groups. We compared the baseline characteristics and clinical outcomes of the external validation set, shown in Supplementary Table S1. The comparison of feature distribution between the training, internal validation, and external validation is shown in in Supplementary Table S2. The incidence rate of SAT in the three groups of patients was similar, and there was no significant difference in age, SOFA score, and initial platelet count in ICU admission.

**Table 2:** Comparisons of baseline characteristics between with thrombocytopenia and without thrombocytopenia.

	**No-SAT (*n* = 723)**	**SAT (*n* = 732)**	**Total (*n* = 1,455)**	***P***
Age (years)	65.6 ± 16.1	65.6 ± 17.1	65.6 ± 16.6	0.97
Male [*n* (%)]	455 (62.9)	471 (64.3)	926 (63.6)	0.613
Alcohol drinking [*n* (%)]	277 (38.3)	262 (35.8)	539 (37)	0.347
Smoking [*n* (%)]	275 (38)	289 (39.5)	564 (38.8)	0.609
CKD [*n* (%)]	17 (2.4)	19 (2.6)	36 (2.5)	0.896
Cancer [*n* (%)]	84 (11.6)	82 (11.2)	166 (11.4)	0.867
Diabetes [*n* (%)]	122 (16.9)	108 (14.8)	230 (15.8)	0.3
Hypertension [*n* (%)]	366 (50.6)	311 (42.5)	677 (46.5)	0.002
APACHE-II	17.1 ± 6.1	19.7 ± 7	18.4 ± 6.7	< 0.001
SOFA	6 ± 2.7	7.8 ± 3.3	6.9 ± 3.2	< 0.001
Sepsis_shock [*n* (%)]	44 (6.1)	145 (19.8)	189 (13)	< 0.001
Antiplatelet drug used [*n* (%)]	185 (25.6)	101 (13.8)	286 (19.7)	< 0.001
**Biochemical indexes on ICU admission**				
Red blood cell (x10^9^/L)	3.8 ± 0.7	3.7 ± 0.8	3.8 ± 0.7	0.038
Hematokrit (L/L)	0.3 ± 0.1	0.3 ± 0.1	0.3 ± 0.1	0.045
White blood cell (x10^9^/L)	11.3 (8.3, 14.77)	12.03 (8.04, 16.71)	11.54 (8.13, 15.39)	0.052
Neutrophil count (x10^9^/L)	9.82 (6.88, 13.19)	10.47 (6.92, 14.93)	10.06 (6.89, 13.93)	0.013
Lymphocyte count (x10^9^/L)	0.83 (0.5, 1.22)	0.69 (0.43, 1.06)	0.75 (0.47, 1.14)	< 0.001
Platelet count (x10^9^/L)	193 (153, 243.5)	211 (154, 274)	201 (153, 256)	0.002
Platelet distribution width (%)	16 (15.5, 16.4)	16.2 (15.8, 16.5)	16.1 (15.7, 16.5)	< 0.001
Mean platelet volume (fl)	9.8 ± 1.3	9.9 ± 1.3	9.8 ± 1.3	0.031
pH	7.42 (7.37, 7.47)	7.38 (7.3, 7.43)	7.4 (7.34, 7.45)	< 0.001
Serum sodium (mmol/L)	140.2 (137.5, 142.8)	141.4 (138.6, 144.1)	140.9 (138, 143.5)	< 0.001
Serum calcium (mmol/L)	2.1 ± 0.2	2 ± 0.2	2 ± 0.2	< 0.001
Serum lactic acid (mmol/L)	1.7 (1.2, 2.6)	3.1 (1.8, 5.2)	2.2 (1.4, 3.85)	< 0.001
Serum bicarbonate (mmol/L)	96 ± 7.3	94.7 ± 8.7	95.4 ± 8	0.002
Prothrombin time(s)	14.4 (13.6, 15.3)	15.4 (14.2, 17.03)	14.8 (13.9, 16.1)	< 0.001
Activated partial thromboplastin time(s)	39.1 (35.4, 44.35)	40.55 (36.07, 47.73)	39.8 (35.7, 46)	< 0.001
International normalized ratio	1.12 (1.05, 1.23)	1.23 (1.12, 1.41)	1.17 (1.08, 1.3)	< 0.001
D-dimer (μg/L)	2.61 (1.28, 5.43)	4.88 (2.21, 12.03)	3.5 (1.58, 8.09)	< 0.001
Alanine aminotransferase (U/L)	20 (13, 37)	24 (15, 55.25)	23 (13, 44)	< 0.001
Aspartate aminotransferase (U/L)	29 (22, 54.5)	45 (26, 99)	36 (23, 70)	< 0.001
Serum albumin (g/L)	32.2 ± 5.1	30.5 ± 5.6	31.3 ± 5.4	< 0.001
C-reactive protein (mg/L)	40 (9.95, 99.85)	62.1 (21.27, 144.92)	55.87 (14.61, 125.15)	< 0.001
Serum urea (mmol/L)	6.92 (5.08, 9.49)	8.08 (5.74, 12.09)	7.53 (5.43, 10.76)	< 0.001
Serum creatinine (mmol/L)	68 (53, 89)	82 (59, 123.25)	74 (56, 105.5)	< 0.001
Procalcitonin (ug/L)	0.41 (0.12, 1.5)	1.04 (0.3, 5.74)	0.67 (0.17, 2.92)	< 0.001

*Continuous variables are described by means and quarterbacks. Categories variables are analyzed by χ^2^ test and continuous variables are analyzed by Wilcoxon rank sum test. SAT, sepsis-associated thrombocytopenia; APACHE, acute physiology and chronic health evaluation; ICU, Intensive Care Unit; CKD, Chronic kidney disease; SOFA, Sepsis-related Organ Failure Assessment*.

**Table 3:** Comparison of infection site and clinical outcomes between groups.

	**No-SAT (*n* = 723)**	**SAT (*n* = 732)**	**Total (*n* = 1,455)**	***P***
Ventilation duration (days)	0.96 (0.28, 5)	3.91 (0.8, 8.8)	2.12 (0.47, 7.38)	<0.001
ICU length of stay (days)	3.88 (1.88, 8.47)	6.97 (3.62, 12.02)	5.22 (2.6, 10.65)	<0.001
Hosp. LOS (days)	19 (13, 29)	18 (11, 28)	19 (12, 28)	0.022
Hospital mortality [*n* (%)]	94 (13)	221 (30.2)	315 (21.6)	<0.001
Cost (x10^3^, yuan)	51.2 (33.5, 79.0)	55.54 (36.3, 87.6)	53.6 (34.5, 82.7)	0.002
**Infection site [*****n*** **(%)]**				
Pulmonary	510 (70.5)	509 (69.5)	1019 (70)	0.718
Urinary	54 (7.5)	78 (10.7)	132 (9.1)	0.043
Blood stream	67 (9.3)	150 (20.5)	217 (14.9)	< 0.001

*Continuous variables are described by means and quarterbacks. Categories variables are analyzed by χ^2^ test and continuous variables are analyzed by Wilcoxon rank sum test. SAT, sepsis-associated thrombocytopenia; ICU, Intensive Care Unit; Hosp. LOS, length of hospital stay*.

### Evaluation of Machine Learning Algorithm Models

**Figure 2:**
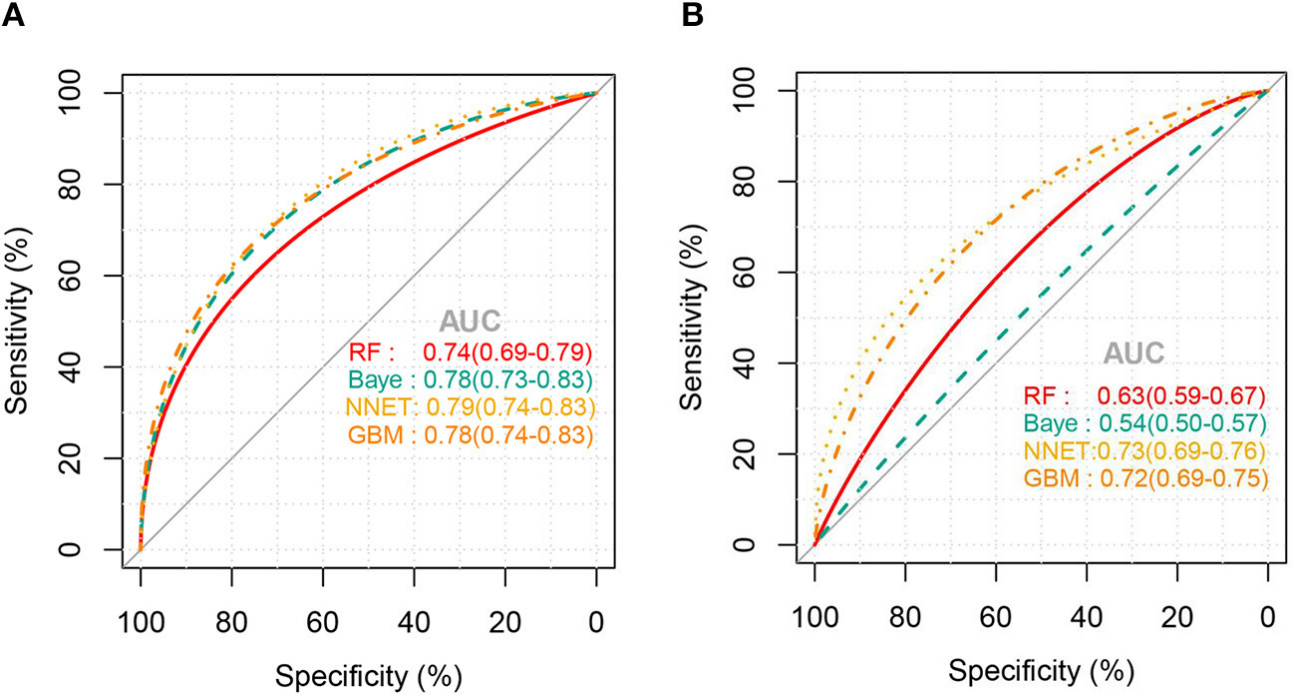
ROC curves of the four machine learning models for predicting thrombocytopenia. **(A)**, Internal validation; **(B)**, external validation; RF, random forest; NNET, neural network; GBM, gradient boosting machine; Baye, bayesian.

**Figure 3:**
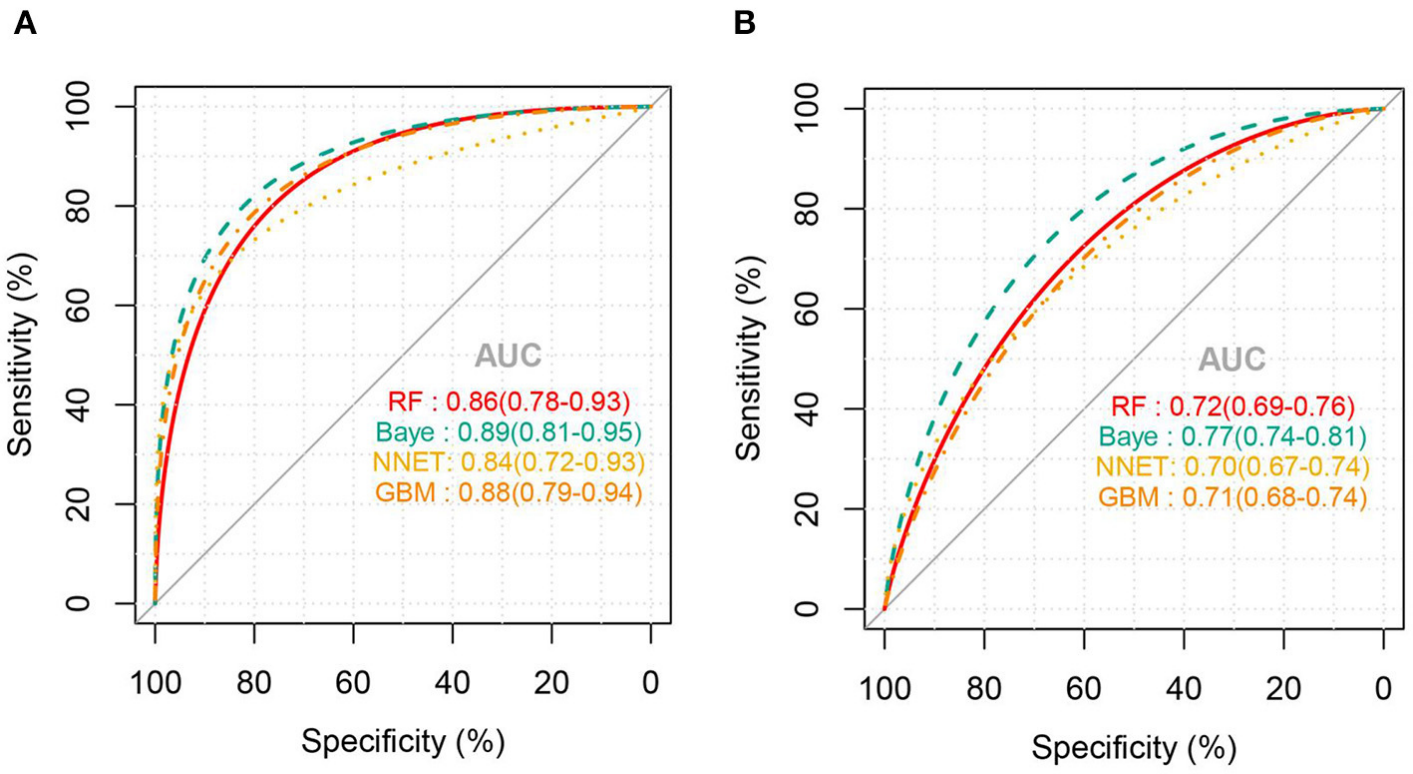
ROC curves of the four machine learning models for predicting severe thrombocytopenia. **(A)**, Internal validation; **(B)**, external validation; RF, random forest; NNET, neural network; GBM, gradient boosting machine; Baye, bayesian.

**Figure 4:**
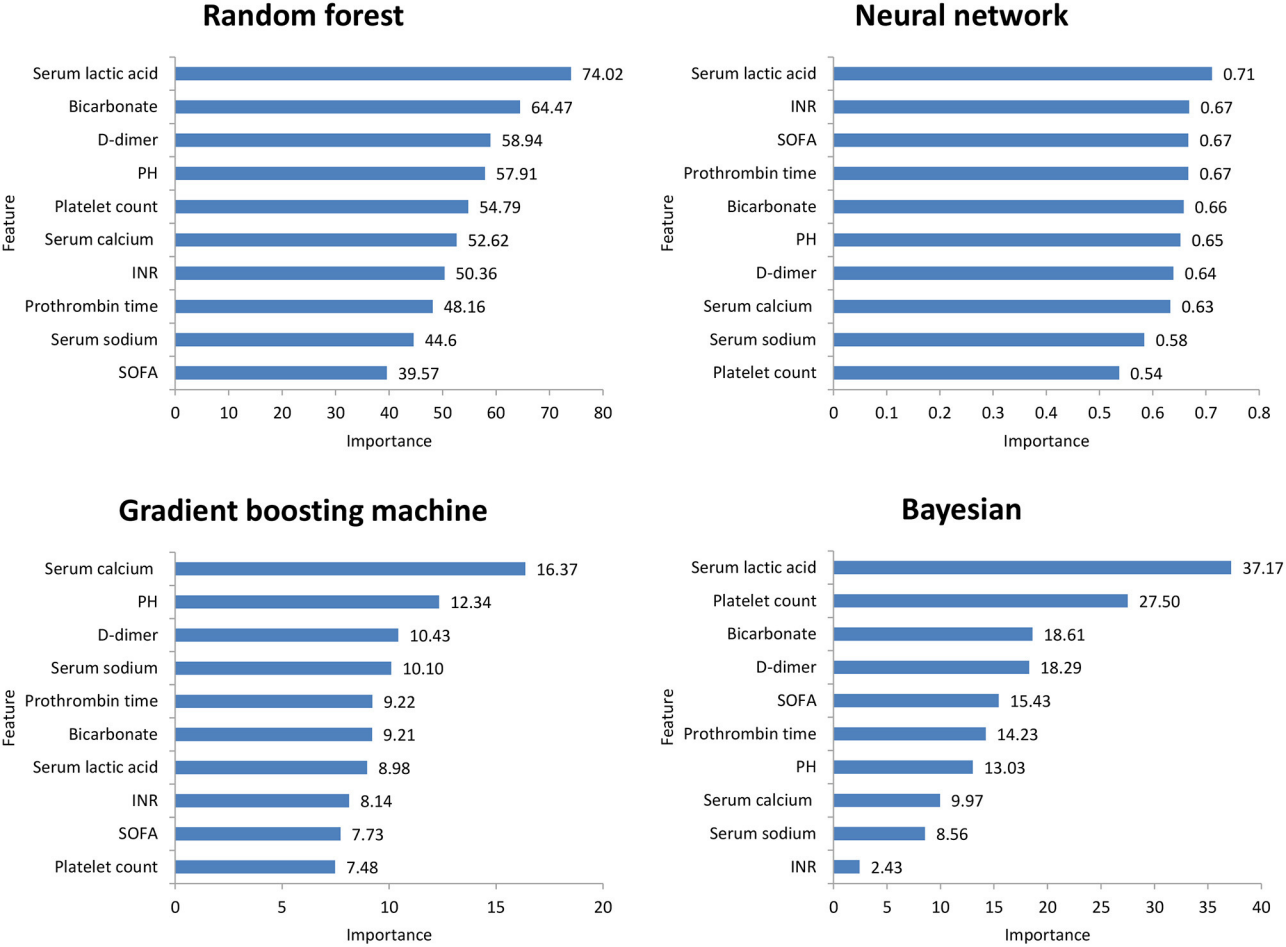
Top 10 variables of the four machine learning models for predicting thrombocytopenia ordered by importance. SOFA, Sepsis-related Organ Failure Assessment; INR, International normalized ratio.

**Figure 5:**
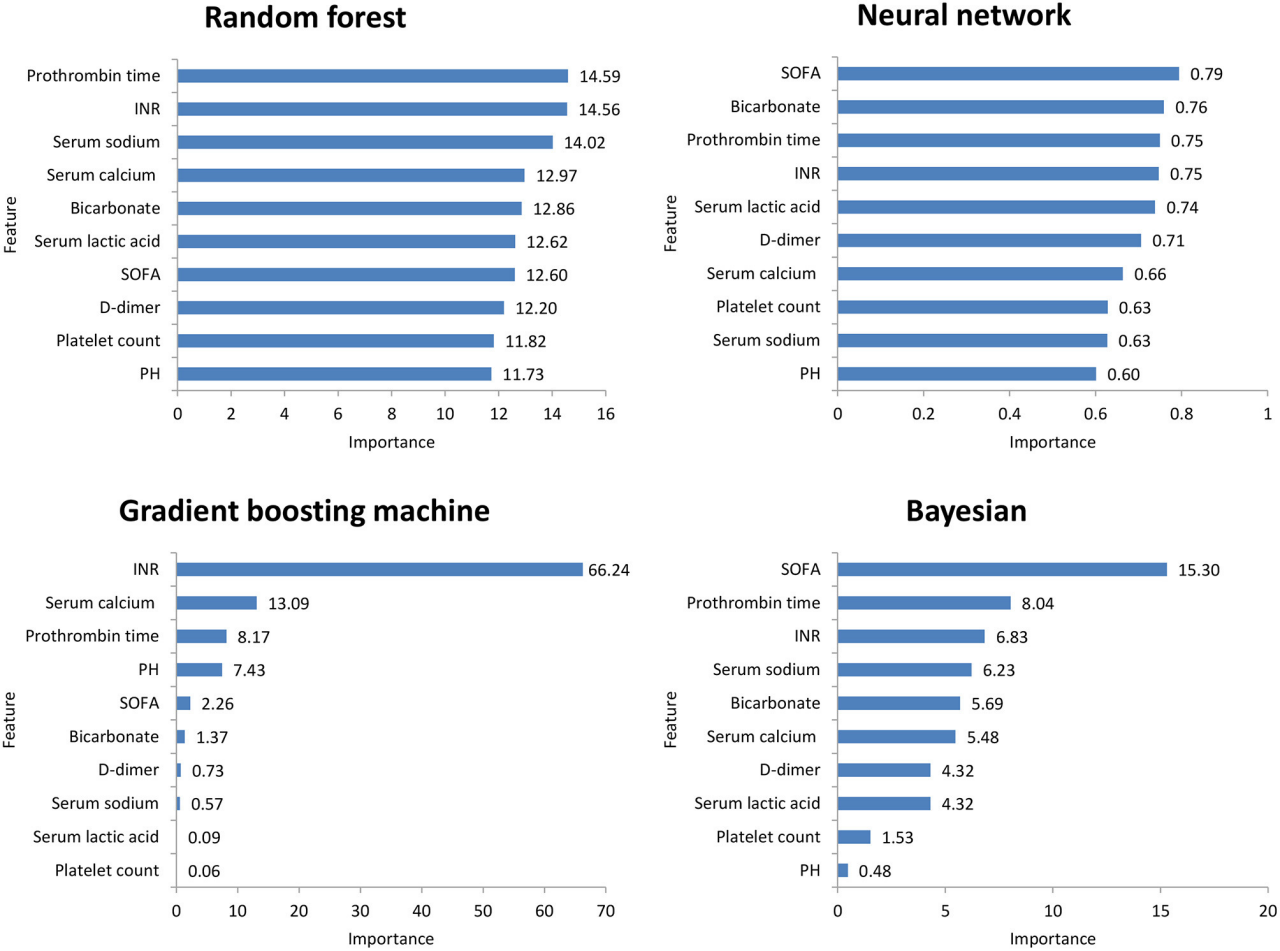
Top 10 variables of the four machine learning models for predicting severe thrombocytopenia ordered by importance. SOFA, Sepsis-related Organ Failure Assessment; INR, International normalized ratio.

Figure 2 shows the ROC comparison of four ML models for thrombocytopenia prediction, with internal validation showing AUCs between 0.74 and 0.79 and external validation showing AUCs between 0.54 and 0.72. Table 3 shows the pairwise comparison in external validation. Results of external validation show that NNET and GBM had the best prediction, with no significant difference between the two models, while the prediction accuracy of RF and Bayesian models was slightly worse. Additional evaluation metrics for the four machine learning models in external validation are presented in Table 4. We established the model for predicting severe thrombocytopenia using the same method. Figure 3 shows the ROC comparison of ML models for the prediction of severe thrombocytopenia, with internal validation showing AUCs between 0.84 and 0.89 and external validation showing AUCs between 0.70 and 0.77. The prediction was better than for thrombocytopenia, with the Bayesian model showing the best results. The calibration curve analysis of models is shown in Supplementary Figure S1. Figures 4, 5 showed the top 10 variables of the four models ordered by importance. LIME provides explanations for any individual patient, and the contribution of a given variable may change depending on other features of the patient in Supplementary Figures S2, S3 shows contributions by the variables for two patients (#2, #3). The red (blue) color indicates that the variable contradicts (supports) a given class.

**Table 4:** Comparison of the area under the roc curve of four machine learning models in external validation.

**Models for predicting thrombocytopenia**				
	**RF**	**Bayesian**	**NNET**	**GBM**
RF	/	0.001	0.001	0.001
Bayesian	0.001	/	0.001	0.001
NNET	0.001		/	0.94
GBM	0.001	0.001	0.94	/
**Models for predicting severe thrombocytopenia**				
	**RF**	**Bayesian**	**NNET**	**GBM**
RF	/	0.001	0.913	0.127
Bayesian	0.001	/	0.001	0.001
NNET	0.913	0.001	/	0.662
GBM	0.127	0.001	0.662	/

*ROC, Receiver operating characteristic; RF, random forest; NNET, neural network; GBM, gradient boosting machine*.

## Discussion

Our study found that SAT had high morbidity and mortality, as well as poor clinical outcomes in ICU, and RF, Bayesian, NNET, and GBM prediction models achieved good predictions.

Thrombocytopenia is very common in ICU patients, with sepsis being its main cause (12). Previous studies on SAT have shown that the incidence rate in critically ill patients (3, 19) was approximately 50%—similar to our findings. Platelets play crucial roles in inflammatory response (20), such as promoting immune response and blood coagulation activation. Presently, many published articles have shown that thrombocytopenia is significantly related to the poor prognosis of patients and is closely related to the degree of thrombocytopenia (21).

Thrombocytopenia is a common reason of platelet transfusion in the ICU. When the platelet count is <50 × 10^9^/ L, clinicians often transfuse platelets (22, 23) to reduce bleeding events. A British prospective multicenter observation study (7) showed that, in ICU patients with severe thrombocytopenia, the mortality rate was as high as 35.4%. Therefore, we also predicted severe thrombocytopenia in patients with sepsis. The models had higher accuracy and better prediction effect. For such patients, early discontinuation of antiplatelet drugs, use of platelet-increasing drugs, and early reservation of platelets might help prevent bleeding events and improve the prognosis of patients.

In this study, among the four ML models, the top variables in terms of importance scores were SOFA score, serum lactic acid, serum sodium bicarbonate, and dimer, which suggested that these factors had a significant correlation with SAT. A retrospective study including 267 patients with abdominal infection showed that a high SOFA score was an important risk factor for hospital-acquired thrombocytopenia. A systematic evaluation (24) found that disease severity was an influencing factor of thrombocytopenia, while serum lactic acid and serum sodium bicarbonate were classic indicators reflecting the severity of the patient's disease. Plasma D-dimer is an important marker of thrombosis activity. In sepsis patients, fibrinolysis activation and D-dimer level have been independently correlated with mortality (25). Therefore, monitoring the SOFA score, serum lactic acid, serum sodium bicarbonate, and dimer levels is helpful for the early detection of thrombocytopenia patients.

This study has some limitations. First, this was a single-center, retrospective study, and some data were missing. We supplemented the data through multiple imputation functions of statistical software to reduce the bias of research results. Second, there are many reasons for thrombocytopenia. For example, some patients with sepsis were treated with hemodialysis, and heparin-induced thrombocytopenia was reported after using heparin. These patients were not excluded, which influenced the results. Third, due to the algorithm characteristics of ML, the models could not clarify the specific relationship between variables and thrombocytopenia, and they were not suitable for all people, which limited the performance of the models. Therefore, based on the algorithms, we showed the measurement of variable importance in the four models and LIME feature plot explained the relationship between variables in the models and thrombocytopenia to a certain extent. Finally, our ML models to predict SAT between ICU stays, the models to predict SAT each day of the ICU stays will be more clinically meaningful. In the future, we will develop software and join the electronic information system to predict SAT each day of the ICU stays.

## Conclusion

We established four ML models to predict SAT and severe thrombocytopenia. The models were validated in MIMIC III and can be used to identify such high-risk patients at an early stage and guide individualized clinical treatment. In the future, we will conduct a prospective cohort study and apply these models to clinical practice.

## Data Availability Statement

The raw data supporting the conclusions of this article will be made available by the authors, without undue reservation.

## Ethics Statement

The studies involving human participants were reviewed and approved by the Ethics Committee of Dongyang People's Hospital. Written informed consent for participation was not required for this study in accordance with the national legislation and the institutional requirements.

## Author Contributions

YW and YP carried out the design and contributed to manuscript revision. XJ participated in data analysis and drafted the manuscript. WZ provided overall supervision and undertook the responsibility of submitting the manuscript for publication. All authors contributed to the article and approved the submitted version.

## Funding

This study was supported by the Clinical Research Fund Project of the Zhejiang Medical Association (2020ZYC-B44 and 2018KY866) and the Conba Hospital Management Project of the Zhejiang Hospital Association (2021ZHA-KEB335).

## Conflict of Interest

The authors declare that the research was conducted in the absence of any commercial or financial relationships that could be construed as a potential conflict of interest.

## Publisher's Note

All claims expressed in this article are solely those of the authors and do not necessarily represent those of their affiliated organizations, or those of the publisher, the editors and the reviewers. Any product that may be evaluated in this article, or claim that may be made by its manufacturer, is not guaranteed or endorsed by the publisher.

## Supplementary Material

Supplementary Figure S1 Calibration of four machine learning models. **(A)**: Models for predicting thrombocytopenia in Internal validation set; **(B)**: Models for predicting thrombocytopenia in external validation set. **(C)**: Models for predicting severe thrombocytopenia in Internal validation set; **(D)**: Models for predicting severe thrombocytopenia in external validation set; RF, random forest; NNET, neural network; GBM, gradient boosting machine; Baye, bayesian.

Supplementary Figure S2 Heatmap plot showing the contribution of each variable to the classification of sample patients. The relative contribution of each variable was calculated using the LIME algorithm. Patients #2, #3 are shown as examples. Red (blue) color indicates that the relevant variable contradicts (supports) a given label. SOFA, Sepsis-related Organ Failure Assessment; INR, International normalized ratio; LIME, Local Interpretable Model-Agnostic Explanations.

Supplementary Figure S3 LIME feature plot shows the contribution of each variable to the classification of sample patients. Red (blue) color indicates that the relevant variable contradicts (supports) a given label. SOFA, Sepsis-related Organ Failure Assessment; INR, International normalized ratio; LIME, Local Interpretable Model-Agnostic Explanations.

Supplementary Table S1 Baseline characteristics and clinical outcomes between SAT and No-SAT groups in the MIMIC III cohort. Continuous variables are described by means and quarterbacks. Categories variables are analyzed by χ^2^ test and continuous variables are analyzed by Wilcoxon rank sum test. SAT, sepsis-associated thrombocytopenia; SOFA, Sepsis-related Organ Failure Assessment; DM, diabetes mellitus; COPD, Chronic Obstructive Pulmonary Disease; AST, aspartate aminotransferase; INR, International normalized ratio; PT, prothrombin time; Hosp. hospital, LOS length of stay; ICU LOS, ICU length of stay.

Supplementary Table S2 Comparison of feature distribution between the training, internal validation, and external validation. Continuous variables are described by means and quarterbacks. Categories variables are analyzed by χ^2^ test and continuous variables are analyzed by Wilcoxon rank sum test. SAT, sepsis-associated thrombocytopenia; ICU, Intensive Care Unit; SOFA, Sepsis-related Organ Failure Assessment; Hosp. LOS, length of hospital stay.

## References

[1] García-Gallo JE Fonseca-Ruiz NJ Celi LA Duitama-Muñoz JF. A machine learning-based model for 1-year mortality prediction in patients admitted to an Intensive Care Unit with a diagnosis of sepsis. Med intensiva. (2020) 44:160–70. 10.1016/j.medin.2018.07.016 30245121

[2] Thorsen-Meyer HC Nielsen AB Nielsen AP Kaas-Hansen BS Toft P Schierbeck J. Dynamic and explainable machine learning prediction of mortality in patients in the intensive care unit: a retrospective study of high-frequency data in electronic patient records. Lancet Digit Health. (2020) 2:e179–91. 10.1016/S2589-7500(20)30018-2 33328078

[3] Sharma B Sharma M Majumder M Steier W Sangal A Kalawar M. Thrombocytopenia in septic shock patients – a prospective observational study of incidence, risk factors and correlation with clinical outcome. Anaesth Intensive Care. (2007) 35:874–80. 10.1177/0310057X0703500604 18084977

[4] Bedet A Razazi K Boissier F Surenaud M Hue S Giraudier S. Mechanisms of thrombocytopenia during septic shock: a multiplex cluster analysis of endogenous sepsis mediators. Shock. (2018) 49:641–8. 10.1097/SHK.0000000000001015 29028771

[5] Xie Y Tian R Xie H Jin W Du J Huang P. The clinical significance of thrombocytopenia complicating sepsis: a meta-analysis. J Infect. (2019) 78:323–37. 10.1016/j.jinf.2018.12.002 30629960

[6] Azkárate I Choperena G Salas E Sebastián R Lara G Elósegui I. Epidemiology and prognostic factors in severe sepsis/septic shock. Evolution over six years. Med Intensiva. (2016) 40:18–25. 10.1016/j.medine.2015.01.002 25746120

[7] Stanworth SJ Walsh TS Prescott RJ Lee RJ Watson DM Wyncoll DL. Thrombocytopenia and platelet transfusion in UK critical care: a multicenter observational study. Transfusion. (2013) 53:1050-8. 10.1111/j.1537-2995.2012.03866.x 22928908

[8] Giannini HM Ginestra JC Chivers C Draugelis M Hanish A Schweickert WD. A machine learning algorithm to predict severe sepsis and septic shock: development, implementation, and impact on clinical practice. Crit Care Med. (2019) 47:1485–92. 10.1097/CCM.0000000000003891 31389839 PMC8635476

[9] Giacobbe DR Signori A Del Puente F Mora S Carmisciano L Briano F. Early detection of sepsis with machine learning techniques: a brief clinical perspective. Front Med. (2021) 8:617486. 10.3389/fmed.2021.617486 33644097 PMC7906970

[10] Collins GS Reitsma JB Altman DG Moons KG. Transparent reporting of a multivariable prediction model for individual prognosis or diagnosis (TRIPOD): the TRIPOD statement. BMJ. (2015) 350:g7594. 10.1161/CIRCULATIONAHA.114.014508 25569120

[11] Johnson A Pollard T Shen L Lehman L Feng M Ghassemi M. MIMIC-III, a freely accessible critical care database. Sci Data. (2016) 3:160035. 10.1038/sdata.2016.35 27219127 PMC4878278

[12] Thiolliere F Serre-Sapin AF Reignier J Benedit M Constantin JM Lebert C. Epidemiology and outcome of thrombocytopenic patients in the intensive care unit: results of a prospective multicenter study. Intensive Care Med. (2013) 39:1460–8. 10.1007/s00134-013-2963-3 23740274

[13] Ben HC Lauzet JY Rézaiguia DS Duvoux C Cherqui D Duvaldestin P. Effect of severe thrombocytopenia on patient outcome after liver transplantation. Intensive Care Med. (2003) 29:756–62. 10.1007/s00134-003-1727-x 12677370

[14] Hui P Cook DJ Lim W Fraser GA Arnold DM. The frequency and clinical significance of thrombocytopenia complicating critical illness: a systematic review. Chest. (2011) 139:271–8. 10.1378/chest.10-2243 21071526

[15] Greinacher A Selleng K. Thrombocytopenia in the intensive care unit patient. Hematology Am Soc Hematol Educ Program. (2010) 2010:135–43. 10.1182/asheducation-2010.1.135 21239783

[16] Singer M Deutschman CS Seymour CW Shankar-Hari M Annane D Bauer M. The third international consensus definitions for sepsis and septic shock (Sepsis-3). JAMA. (2016) 315:801–10. 10.1001/jama.2016.0287 26903338 PMC4968574

[17] Zhang Zh Chen L Xu P Hong YC. Predictive analytics with ensemble modeling in laparoscopic surgery: A technical note. Laparosc Endosc Robot Surg. (2022). 10.1016/j.lers.2021.12.003

[18] Zhang Z Gayle AA Wang J Zhang H Cardinal FP. Comparing baseline characteristics between groups: an introduction to the CBCgrps package. Ann Transl Med. (2017) 5:484. 10.21037/atm.2017.09.39 29299446 PMC5750271

[19] Venkata C Kashyap R Farmer JC Afessa B. Thrombocytopenia in adult patients with sepsis: incidence, risk factors, and its association with clinical outcome. J Intensive Care. (2013) 1:9. 10.1186/2052-0492-1-9 25810916 PMC4373028

[20] Vardon BF Ruiz S Gratacap MP Garcia C Payrastre B Minville V. Platelets are critical key players in sepsis. Int J Mol Sci. (2019) 20:3494. 10.3390/ijms20143494 31315248 PMC6679237

[21] Vandijck DM Blot SI De WJ Hoste EA Vandewoude KH Decruyenaere JM. Thrombocytopenia and outcome in critically ill patients with bloodstream infection. Heart Lung. (2010) 39:21–6. 10.1016/j.hrtlng.2009.07.005 20109983

[22] Ning S Barty R Liu Y Heddle NM Rochwerg B Arnold DM. Platelet transfusion practices in the ICU: data from a large transfusion registry. Chest. (2016) 150:516–23. 10.1016/j.chest.2016.04.004 27102183

[23] Arnold DM Crowther MA Cook RJ Sigouin C Heddle NM Molnar L. Utilization of platelet transfusions in the intensive care unit: indications, transfusion triggers, and platelet count responses. Transfusion. (2006) 46:1286–91. 10.1111/j.1537-2995.2006.00892.x 16934061

[24] Jonsson AB Rygård SL Hildebrandt T Perner A Møller MH Russell L. Thrombocytopenia in intensive care unit patients: a scoping review. Acta Anaesthesiol Scand. (2021) 65:2–14. 10.1111/aas.13699 32916017

[25] Semeraro F Colucci M Caironi P Masson S Ammollo CT Teli R. Platelet drop and fibrinolytic shutdown in patients with sepsis. Crit Care Med. (2018) 46:e221–8. 10.1097/CCM.000000000000291 29261568

