# Supplementary figures and images for: Prediction Models for Sepsis-Associated Thrombocytopenia Risk in Intensive Care Units Based on a Machine Learning Algorithm
Source: Front Med (Lausanne). 2022 Jan 27;9:837382. doi: 10.3389/fmed.2022.837382 (PMC8829034; doi:10.3389/fmed.2022.837382)

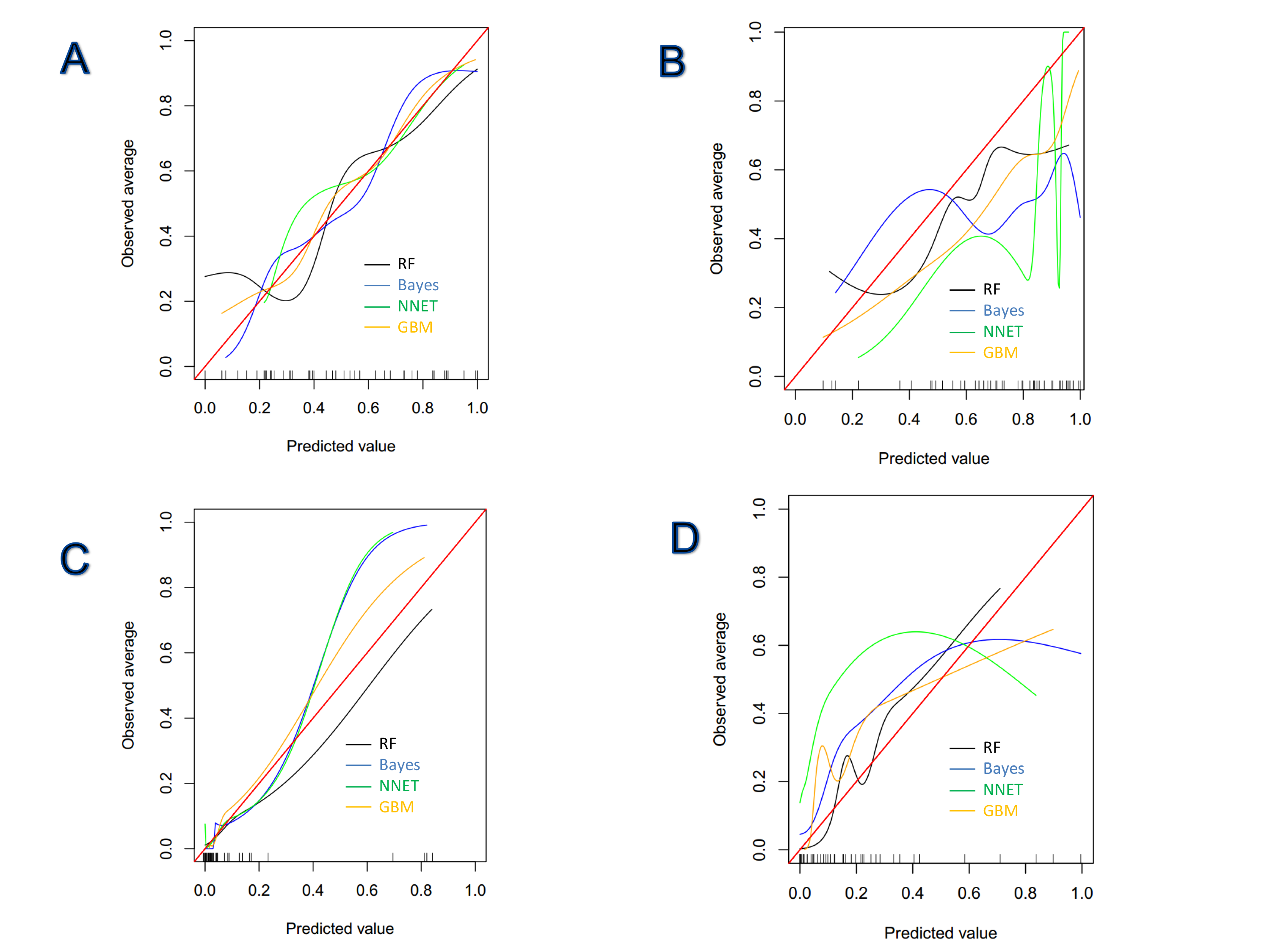

Supplement: Supplementary Figure S1 — Calibration of four machine learning models. (A): Models for predicting thrombocytopenia in Internal validation set; (B): Models for predicting thrombocytopenia in external validation set. (C): Models for predicting severe thrombocytopenia in Internal validation set; (D): Models for predicting severe thrombocytopenia in external validation set; RF, random forest; NNET, neural network; GBM, gradient boosting machine; Baye, bayesian. [file Image_1.TIF]

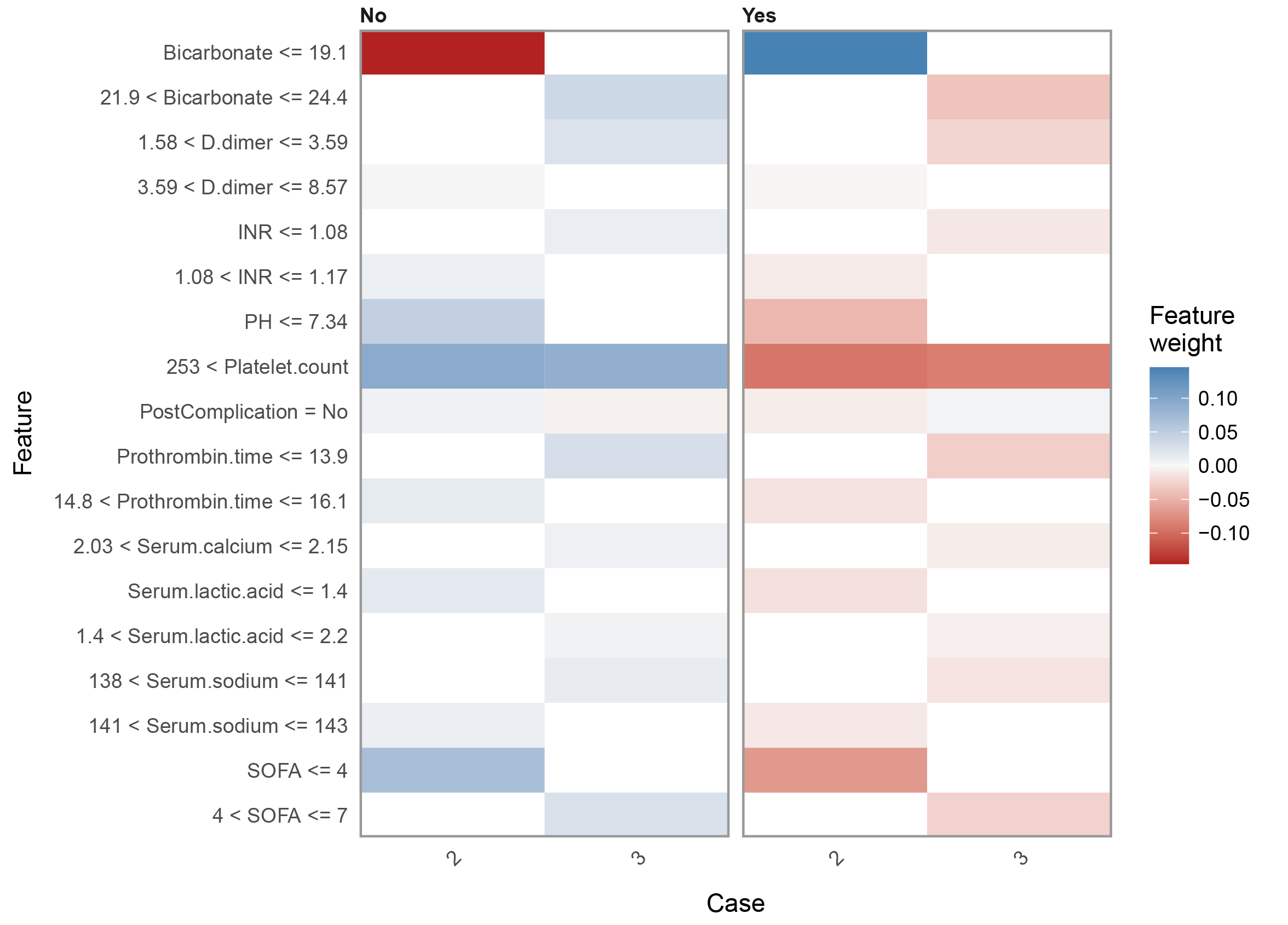

Supplement: Supplementary Figure S2 — Heatmap plot showing the contribution of each variable to the classification of sample patients. The relative contribution of each variable was calculated using the LIME algorithm. Patients #2, #3 are shown as examples. Red (blue) color indicates that the relevant variable contradicts (supports) a given label. SOFA, Sepsis-related Organ Failure Assessment; INR, International normalized ratio; LIME, Local Interpretable Model-Agnostic Explanations. [file Image_2.TIF]

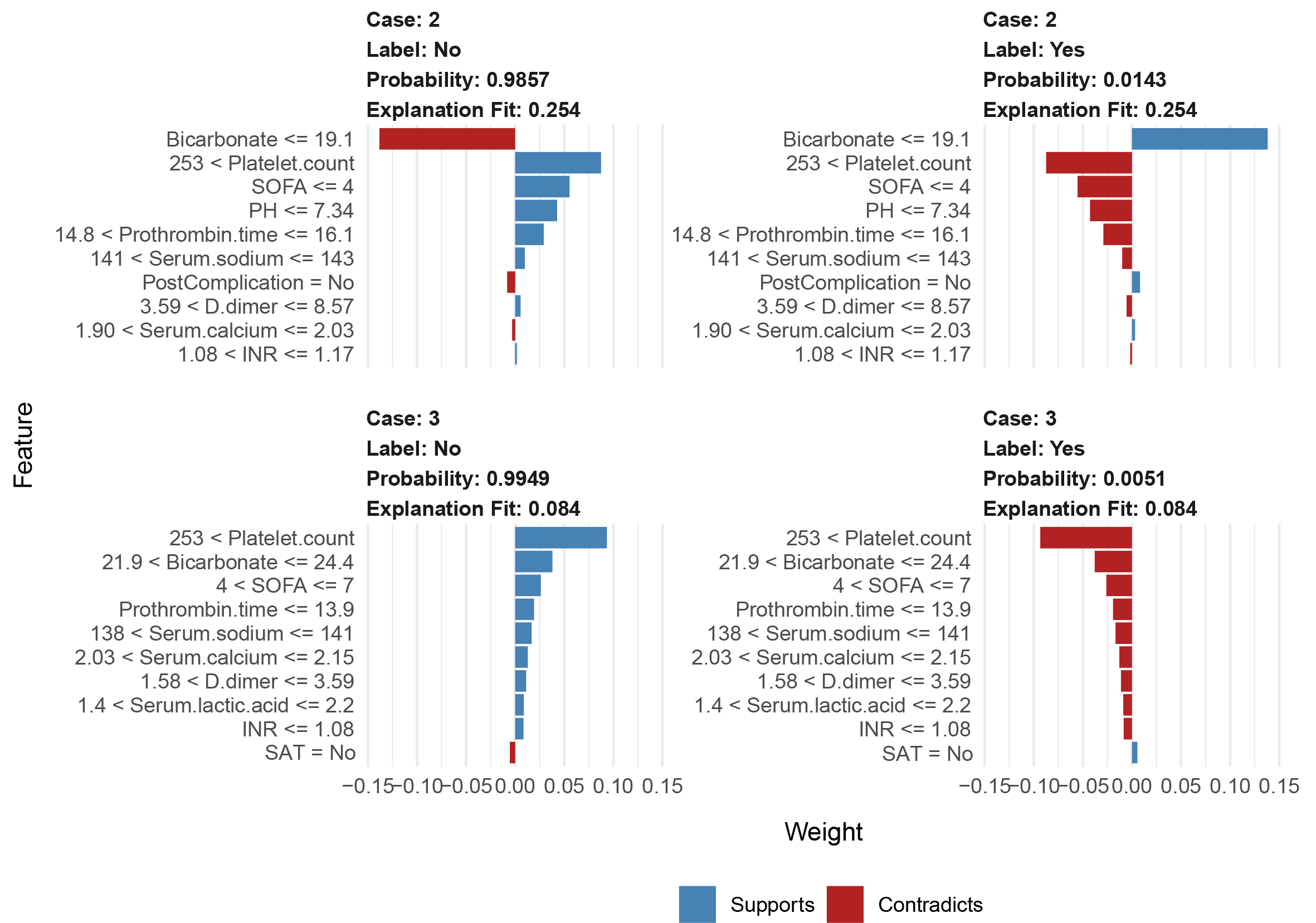

Supplement: Supplementary Figure S3 — LIME feature plot shows the contribution of each variable to the classification of sample patients. Red (blue) color indicates that the relevant variable contradicts (supports) a given label. SOFA, Sepsis-related Organ Failure Assessment; INR, International normalized ratio; LIME, Local Interpretable Model-Agnostic Explanations. [file Image_3.TIF]
