# Supplementary material for: Prediction Models for Sepsis-Associated Thrombocytopenia Risk in Intensive Care Units Based on a Machine Learning Algorithm
Source: Front Med (Lausanne). 2022 Jan 27;9:837382. doi: 10.3389/fmed.2022.837382 (PMC8829034; doi:10.3389/fmed.2022.837382)
Supplement: Supplementary Table S1 — Baseline characteristics and clinical outcomes between SAT and No-SAT groups in the MIMIC III cohort. Continuous variables are described by means and quarterbacks. Categories variables are analyzed by χ2 test and continuous variables are analyzed by Wilcoxon rank sum test. SAT, sepsis-associated thrombocytopenia; SOFA, Sepsis-related Organ Failure Assessment; DM, diabetes mellitus; COPD, Chronic Obstructive Pulmonary Disease; AST, aspartate aminotransferase; INR, International normalized ratio; PT, prothrombin time; Hosp. hospital, LOS length of stay; ICU LOS, ICU length of stay. [file Table_2.DOCX]

Table S1 Baseline Characteristics and Clinical Outcomes between SAT and No-SAT Groups in the MIMIC Ⅲ Cohort

| Variables | | SAT (n=364) | no-SAT (n=324) | P-Value |
| --- | --- | --- | --- | --- |
| Demographic variables | |  |  |  |
|  | Age (years) | 66.75 (53.94, 77.62) | 67.64 (53.34, 78.9) | 0.69 |
|  | Male [n(%)] | 201 (55.2) | 174 (53.7) | 0.748 |
|  | SOFA | 8 (5, 10) | 5 (3, 7) | <0.001 |
| Admission type [n(%)] | |  |  | 0.006 |
|  | Elective | 22 (6) | 6 (1.9) |  |
|  | Emergency | 324 (89) | 309 (95.4) |  |
|  | Urgent | 18 (4.9) | 9 (2.8) |  |
| Comorbidities [n(%)] | |  |  |  |
|  | Hypertension | 101 (27.7) | 101 (31.2) | 0.368 |
|  | DM | 87 (23.9) | 92 (28.4) | 0.21 |
|  | COPD | 7 (1.9) | 8 (2.5) | 0.82 |
|  | Coronary | 44 (12.1) | 41 (12.7) | 0.913 |
| Laboratory tests | |  |  |  |
| Creatinine(mmol/L) | | 1.4 (0.9, 2.43) | 1.2 (0.8, 2.02) | 0.031 |
| Urea(mmol/L) | | 30.5 (19, 51.25) | 27 (15, 42) | 0.001 |
| AST(IU/L), | | 59 (29, 193.25) | 32 (22, 65.25) | < 0.001 |
| D-dimer (μg/L) | | 2.06 (1, 5.28) | 1.71 (0.89, 3.15) | < 0.001 |
|  | INR, | 1.6 (1.3, 2.1) | 1.5 (1.3, 1.9) | 0.064 |
|  | PT (s), | 16.1 (14.2, 19.4) | 15.8 (14, 18.13) | 0.147 |
|  | Platelet (×109/L), | 193 (137.75, 294) | 210 (153.75, 281) | 0.179 |
|  | WBC (×109/L), | 14.65 (9.47, 20.7) | 13 (8.58, 19.6) | 0.071 |
|  | PH | 7.3 ± 0.1 | 7.4 ± 0.1 | < 0.001 |
|  | Bicarbonate (mmol/L) | 20.2 ± 5.7 | 21.5 ± 4.9 | 0.001 |
|  | Calcium (mmol/L) | 7.8 (7.1, 8.42) | 7.8 (7.2, 8.3) | 0.891 |
|  | Potassium (mmol/L) | 4.2 ± 0.8 | 4.1 ± 0.8 | 0.169 |
|  | Sodion (mmol/L) | 138.5 ± 5.7 | 138.5 ± 5.1 | 0.975 |
|  | Lactate (mmol/L) | 2.4 (1.6, 4.2) | 1.6 (1.2, 2.3) | < 0.001 |
|  | Glucose (mmol/L) | 131.5 (102.75, 173.25) | 127 (104.75, 166) | 0.685 |
| Outcome | |  |  |  |
|  | Hosp. LOS (day) | 18.35 (9.55, 30.8) | 10.79 (6.03, 19.69) | <0.001 |
|  | ICU LOS (day) | 9.48 (5.08, 19.98) | 3.07 (1.99, 6.21) | <0.001 |
|  | Hospital mortality [n(%)] | 149 (40.9) | 58 (17.9) | <0.001 |

Abbreviations: Continuous variables are described by means and quarterbacks. Categories variables are analyzed by χ2 test and continuous variables are analyzed by Wilcoxon rank sum test. SAT: sepsis-associated thrombocytopenia; SOFA: Sepsis-related Organ Failure Assessment. DM: [diabetes](javascript:;) [mellitus](javascript:;); COPD: Chronic Obstructive Pulmonary Disease; AST: aspartate aminotransferase; INR: International normalized ratio. PT: prothrombin time; Hosp. hospital: LOS length of stay; ICU LOS: ICU length of stay
