# Supplementary material for: Prediction Models for Sepsis-Associated Thrombocytopenia Risk in Intensive Care Units Based on a Machine Learning Algorithm
Source: Front Med (Lausanne). 2022 Jan 27;9:837382. doi: 10.3389/fmed.2022.837382 (PMC8829034; doi:10.3389/fmed.2022.837382)
Supplement: Supplementary Table S2 — Comparison of feature distribution between the training, internal validation, and external validation. Continuous variables are described by means and quarterbacks. Categories variables are analyzed by χ2 test and continuous variables are analyzed by Wilcoxon rank sum test. SAT, sepsis-associated thrombocytopenia; ICU, Intensive Care Unit; SOFA, Sepsis-related Organ Failure Assessment; Hosp. LOS, length of hospital stay. [file Table_3.DOCX]

Table S2 Comparison of feature distribution between the training, internal validation, and external validation

| **Variables** | **Training (n=1092)** | **Internal validation (n=363)** | **External validation**  **(n=688)** | **P value** |
| --- | --- | --- | --- | --- |
| Age (years) | 67.5 (54, 78) | 70 (55, 80) | 67.2 (53.58, 78.15) | 0.121 |
| Male [n(%)] | 688 (63) | 238 (66) | 375 (55) | < 0.001 |
| SOFA | 7 (4, 9) | 7 (4, 9) | 6 (4, 9) | 0.176 |
| Comorbidities [n(%)] |  |  |  |  |
| Hypertension | 506 (46) | 171 (47) | 202 (29) | < 0.001 |
| Diabetes | 162 (15) | 68 (19) | 179 (26) | < 0.001 |
| Biochemical indexes on ICU admission |  |  |  |  |
| Platelet count (x10^9/L) | 201.5 (153, 257) | 199 (154, 255.5) | 203 (146, 286.25) | 0.486 |
| pH | 7.4 (7.34, 7.45) | 7.39 (7.33, 7.45) | 7.35 (7.27, 7.4) | < 0.001 |
| Serum bicarbonate (mmol/L) | 21.8 (18.8, 24.3) | 22.3 (19.35, 24.8) | 21 (17, 24) | < 0.001 |
| Serum lactic acid(mmol/L) | 2.2 (1.4, 3.9) | 2.1 (1.35, 3.8) | 2 (1.4, 3.3) | 0.009 |
| Outcome |  |  |  |  |
| SAT [n(%)] | 557 (51) | 175 (48) | 364 (53) | 0.347 |
| ICU length of stay (days) | 5.26 (2.61, 10.76) | 5.06 (2.55, 9.92) | 5.94 (2.73, 13.18) | 0.014 |
| Hosp. LOS (days) | 19 (12, 28) | 18 (12, 28) | 14.02 (7.92, 26.72) | < 0.001 |
| Hospital mortality [n(%)] | 227 (21) | 88 (24) | 207 (30) | < 0.001 |

Abbreviations: Continuous variables are described by means and quarterbacks. Categories variables are analyzed by χ2 test and continuous variables are analyzed by Wilcoxon rank sum test. SAT: sepsis-associated thrombocytopenia; ICU Intensive Care Unit; SOFA: Sepsis-related Organ Failure Assessment: Hosp. LOS: length of hospital stay.
